# The state of decentralization of the healthcare system and nutrition programs in the Lao People’s Democratic Republic: an organizational study

**DOI:** 10.1186/s12913-024-11513-y

**Published:** 2024-09-06

**Authors:** Phonevilay Viphonephom, Sengchanh Kounnavong, Daniel Reinharz

**Affiliations:** 1 Department of Social and Preventive Medicine, Laval University, Quebec City, QC Canada; 2 Lao Tropical and Public Health Institute (Lao TPHI, Ministry of Health, Vientiane, Lao PDR

**Keywords:** Decentralization, Nutrition, Neo-institutional Theory, Single-party state, Lower-middle income country, Lao PDR

## Abstract

**Background:**

The Lao People’s Democratic Republic (Lao PDR), a lower-middle income country, has a higher malnutrition rate than other Southeast Asian countries. The decentralization of healthcare is a determinant of the effectiveness of programs to reduce malnutrition, but no study has focused on this factor in this country. This organizational study explores the state of decentralization of the healthcare system in Lao PDR that underlies the nutrition programs in the country.

**Methods:**

A qualitative study, which is based on a neo-institutional theory conceptual framework, explored factors related to dominant structure (laws, regulations, resources) and interpretative schemes (dominant ideas and beliefs) that characterize the nutrition services provided in the Lao healthcare system. Twenty-four semistructured interviews were performed with representatives of healthcare institutions involved in nutrition programs at different government levels, external donors and civil society organizations. The interviews were completed with relevant documents. The analysis focused on the convergence of interpretative schemes of the organizations concerned and the coherence between the structure underpinning the nutrition programs and the interpretative schemes.

**Results:**

Services deployed to reduce malnutrition in the Lao PDR remain largely centralized, despite factors specific to the country that led it to promote decentralization of its services. The convergence of interpretive schemes and the coherence between the observed structure and the interpretative schemes of actors at all governance levels ensure the stability of this state of decentralization, which has persisted for almost 50 years.

**Conclusion:**

Nutrition programs in the Laos PDR are largely under the responsibility of the central government. The transformations in the healthcare system, notably with the use of new information technologies and the fact that the provinces are populated by a growing number of professionals trained in nutrition in addition to factors that push the system to be decentralized, such as ethnic diversity, the increasing availability of human resources in provinces, and the use of communication technologies, are not strong enough to change the balance of power between governance levels. The *deconcentration* that characterizes decentralization is therefore likely to continue for the foreseeable future.

## Background

The World Health Organization (WHO) defines malnutrition as deficiencies, excesses, or imbalances in a person’s intake of energy and/or nutrients, encompassing three broad groups of conditions: undernutrition (including wasting, stunting, and underweight); micronutrient-related malnutrition (including deficiencies or excesses of essential vitamins and minerals); and overweight, obesity, and diet-related noncommunicable diseases [1]. Undernutrition and micronutrient-related malnutrition are the main issues generally addressed by nutrition interventions aimed at five-year-old children in developing countries [2, 3]. Children under 5 in lower-middle-income countries are particularly vulnerable to undernutrition and its lifelong effects [4, 5], which manifest as wasting (acute undernutrition or severe weight loss due to insufficient food intake and/or infectious disease), stunting (chronic undernutrition leading to low height-for-age and irreversible long-term physical and cognitive damage), and underweight (low weight-for-age, potentially indicating stunting, wasting, or both) [5]. The WHO highlights that nearly half of deaths among children under 5 years of age are linked to undernutrition. These deaths predominantly occur in low-and middle-income countries [1].

Undernutrition among children under five years of age is a public health priority in the Lao PDR [6]. The prevalence of stunting, an indicator often used to estimate the prevalence of chronic malnutrition, is among the highest in East Asia [7], with a national rate estimated at 31.5% in 2022 [8]. This rate surpasses those in neighboring lower-middle-income countries, such as Vietnam (20%), Cambodia (22%), and Myanmar (27%) [9]. According to the WHO standards, stunting is considered critical if it exceeds the threshold of 30% [5]. Acute malnutrition, estimated by the prevalence of wasting, is still a public health problem among children under five years of age in Lao PDR. The prevalence of wasting is 10%, which is the same as that in Cambodia but higher than that in Myanmar (7%) and Vietnam (5%) [9]. Furthermore, the problem of malnutrition is unevenly distributed across the territory of Lao PDR. Rural and ethnic minority children are at greater risk of stunting than are urban and majority ethnic groups [10]. There was a 15% lower risk of stunting for urban children than for their rural counterparts (24% vs. 39%). In Phongsaly Province, the stunting prevalence is as high as 54%, in contrast to the 14% reported in the Vientiane Capital. Additionally, the prevalence of wasting was the lowest in the province of Luangnamtha (3%) and highest in the province of Xayabury (19%) [11].

The state of healthcare system decentralization has been shown to be a determinant of nutritional status in developing countries [12, 13]. Decentralization involves transferring power and responsibility from the central government to lower levels of governance, impacting healthcare service accessibility, quality, and equity [14–17]. Decentralization is commonly categorized into three major forms: 1) Deconcentration involves the central government handing over some authority to local administrative offices within the health ministry, allowing local management to handle health-related activities with some discretion; 2) Delegation entails the central government transferring defined managerial and administrative functions to institutions outside the central government's hierarchical structure, which are indirectly controlled by the health ministry; and 3) Devolution refers to the central government legally transferring power to locally elected political organs that operate independently of the central government in specific functions [15].

Lao PDR *has a deconcentration* form of decentralization [18, 19]. While local levels can provide input for program adaptation to local needs, ultimate control remains with the central government, which can veto decisions made by regional or local health offices [20, 21]. In Lao PDR, the dynamics and forces influencing the current state of decentralization are shaped by the country's unique historical-political context and distinctive population characteristics. These factors contribute to the complex nature of power sharing across different government levels. The first factor is related to the historical concept of Lao PDR, which is rooted in the notion of "*meuang*"[22]. A *meuang* is a walled city led by a local ruler overseeing surrounding settlements and villages [22, 23]. Each *meuang* maintained its governance, with oversight and protection from the kingdom but without interference in internal affairs. While the nineteenth century shift toward a centralized state model introduced Western administrative practices, the *meuang* concept remains deeply ingrained in Lao society, lending historical legitimacy to decentralization forces [22, 24]. The second factor is the country's ethnic diversity. The country has 49 official minority groups constituting nearly half the population. Minorities often reside in remote areas [25]. Decentralizing power and assigning responsibilities to local levels is expected to empower authorities to more effectively customize health programs to meet the diverse needs of the population.

These decentralizing forces are counterbalanced by centralizing forces due in great part to the sociocultural context influenced by Confucianist ideology, which emphasizes social harmony and respect for authority and promotes political centralization in East Asia, including Lao PDR [26–28]. Moreover, the limited human and financial resources of the central government, which still rely heavily on donor funding, push toward the centralization of the healthcare system and program functioning [29–31]. From an organizational perspective, these factors create a complex balance between decentralization and centralization in the Lao healthcare system.

Little work has been done in Lao PDR on how the state of decentralization affects the functioning of public health programs, despite being an organizational determinant of health. This organizational study examines the status of decentralization of the healthcare system in Lao PDR and its influence on the National Nutrition Policy.

## Methods

### Conceptual framework

The conceptual framework used in this study was based on neo-institutional theory (NIT) [32, 33]. NITs view organizations as social structures that must conform to the expectations and pressures of their institutional environment, which includes normative and regulatory forces [34, 35]. This approach was considered relevant for this study because decentralization is an institutionalized concept. The form of decentralization that underlies the nutrition policy in the country arises from sociohistorical and legal factors that institutionalize the distribution of powers that govern the functioning of the policy.

Different NIT conceptual frameworks have been proposed. The one used in this study is the one that was proposed by Hinings and Greenwood in 1988 [36]. It considers the dialectic between the structure, i.e., the laws, rules, and resources that constrain the work performed in an organization, and the interpretive schemes and their meaning-making processes that shape individual and collective behavior that are dominant in a society, i.e., the dominant ideas and beliefs [36, 37]. This dialectic approach has been widely used in studies on public policies concerning the nature of institutional structures [38–42]. From these two dimensions, the structural dimension emphasizes the importance of formalizing corporate values and standards in the organization’s standardized policies and procedures, formalizing decision-making processes, and creating hierarchical structures to ensure compliance with institutionalized environmental factors. On the other hand, the interpretive schemes dimension focuses on the importance of shared meanings and beliefs in shaping organizational behaviors [32, 43]. This dimension emphasizes that organizations are influenced by both individual and shared beliefs, values, and norms within their institutional environment. These elements shape the organizational actors' understanding and interpretation of what constitutes legitimate and appropriate behaviors [35, 44, 45]. By doing so, organizations can gain legitimacy and ensure their survival in complex and dynamic institutional environments [34, 35, 45, 46].

### Study design

A case study based on an ethnographic design was conducted to examine patterns of social organization and ideational systems that underlie formal/informal structures, as well as the dominant values, behaviors and beliefs of the groups involved in nutrition interventions in Lao PDR [47–49]. The data were collected from documents and semistructured interviews with representatives of governmental organizations, nongovernmental organizations (NGOs), and civil society organizations (CSOs) involved in nutrition programs in four provinces of Lao PDR. The nutrition programs in this study are those that align with the Lao National Nutrition Plans and Strategies; they encompass interventions and programs from the health and other sectors that are expected to positively influence nutritional indicators. The data were collected between January and June 2022.

### Data collection

#### Interview Guide

**Fig. 1:**
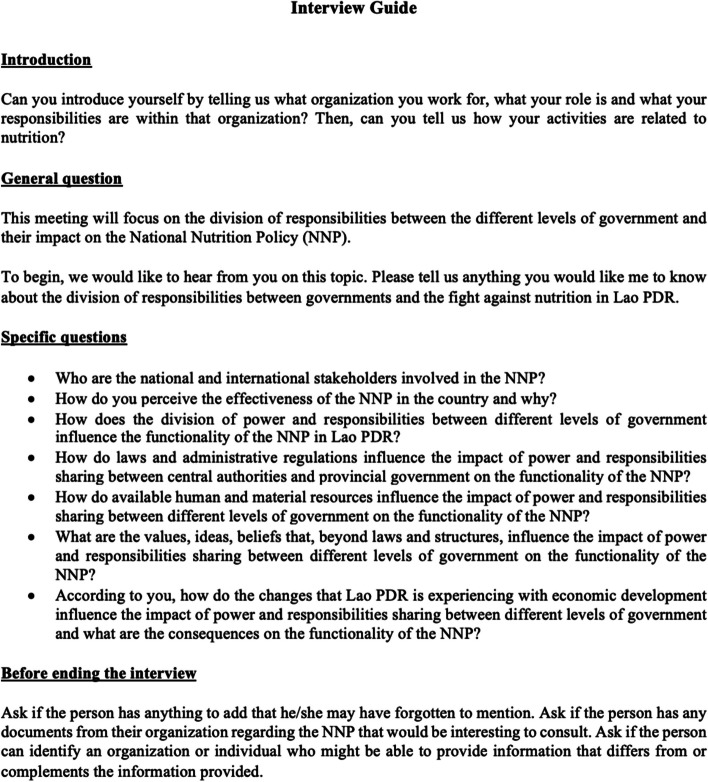
Interview Guide

An interview guide (see Fig. 1) was developed for this study based on the dimensions of the conceptual framework. This interview guide included a pretest with three members of the Lao Tropical and Public Health Institute (Lao TPHI), who has experience in nutrition research in Lao PDR. These individuals were not part of the study's participants. The participants were asked about their current state of malnutrition in Lao PDR and changes over time, as well as insights into power sharing among government levels and external actors. The questions also covered the organization’s experiences and responsibilities in the field of nutrition. Discussions have further explored perspectives on structural elements (policies, regulations, rules) and common beliefs affecting the healthcare system's ability to address nutrition issues.

#### Study sites

To capture the diversity of the country, the latest available national-level data on nutritional status were presented for three provinces with contrasting nutrition situations: Luangnamtha, Xayabury, and Saravan (Table 1). Vientiane capital was chosen because of its status as the location of the Ministry of Health (MoH) and national child nutrition-focused institutions (Maternal and Child Health Center and Nutrition Center), as well as the headquarters of development donors and civil society organizations. Luangnamtha, a northern province and one of the most economically developed in the country, has seen remarkable improvements in nutrition indicators. Xayabury, which is also in the north and adjacent to the capital, has experienced little improvement in terms of stunting and underweight rates, with an increase in wasting in recent years. Saravan, in the southern region and one of the poorest provinces of the country, has among the highest prevalence rates of all types of malnutrition, with no significant changes in recent years [50, 51].

**Table 1:** Percentage of children under age 5 by nutritional status according to three anthropometric indices: weight for age, height for age, and weight for height [50, 51]

Study sites	Stunting (percent below -2 SD^*^)		Wasting (percent below -2 SD^*^)		Underweight (percent below -2 SD^*^)	
	**2012**	**2017**	**2012**	**2017**	**2012**	**2017**
**Luangnamtha**	53	34	21	3	40	19
**Xayabury**	39	25	6	19	23	19
**Saravan**	54	43	9	13	41	29

^*^-2 *SD* Standard deviation (SD) considered as moderate and severe

#### Source of information

Two sources of information were used for the analysis in this study: interviews with representatives of key institutions and relevant documents [52].

##### Representatives of key institutions

The following criteria were used to recruit participants for individual interviews. The participants had to 1) occupy a formal position in relation to nutrition within their organization; 2) be in their current position for at least one year; 3) be authorized to speak on behalf of their organization regarding projects related to nutrition in children under five years of age; and 4) be able to speak one of the languages spoken by the interviewer: Lao (official language), English, or French.

A purposeful sampling approach was used to constitute the sample [53, 54]. A preliminary list of participants was created by one of the researchers (SK). At the end of each interview, the participants were asked to suggest additional organizations or individuals who could offer different perspectives. This snowball sampling method aimed to diversify opinions until data saturation was achieved [47, 55].

Following administrative protocols, a project summary and discussion topics were initially sent to organization directors for approval. The latter identified individuals within their organization who might have participated in the study. These persons were contacted and asked for their permission to receive project details via fax, WhatsApp, or email. Any queries were addressed through phone calls. If the solicited person agreed, they signed a consent form. The interviews were subsequently scheduled at a mutually convenient time. The participants could choose between face-to-face or online videoconference interviews (Zoom or Google Meet). The interviews, which lasted 45 to 90 min, were audio-recorded with consent, and handwritten notes were taken for documentation and impressions.

##### Documents

To supplement the information provided during the interviews, all documents written in English, Lao, or French on nutrition policies and power sharing between different government levels in Lao PDR were also analyzed. These documents included published academic papers, documents provided by individuals who were interviewed, and documents found on the website of their organization.

### Data analysis

All interviews conducted in Lao or French were translated into English by the interviewer and sent to the coauthor who has the least mastery of the Lao language. The realism of the translation was verified by sending a first version of the analyses to three people interviewed, who had to confirm that they found their opinions there. The interviews and document content were analyzed using NVivo 11 software. The interviews and document contents were subsequently analyzed through four main steps: 1) data coding (where data were segmented into meaningful units such as phrases, sentences, or paragraphs); 2) categorization (coded data were then grouped into themes or concepts); 3) coding of themes in connection with the conceptual framework; and 4) comparison of emerging information with published studies on the topic [56].

The analysis was performed using an inductive-deductive approach [57, 58]. The validity of the results was ensured by four elements: 1) credibility based on the triangulation of information provided by multiple sources; 2) transferability through a detailed description of the participants, research process and study context to help the reader judge the relevance of the results for another context [59, 60]; 3) reliability based on analyses performed independently by the researcher and one of her supervisors and the search for a consensus between them in case of discrepancy; and 4) confirmability through a detailed and transparent record of the research process, including the data collection, analysis, interpretation and discussion notes from the meetings with coresearchers [61].

## Results

Twenty-four semistructured interviews were conducted with representatives of key organizations: government institutions, United Nations (UN) agencies, international nongovernmental organizations (NGOs), and civil society organizations (CSOs). The participants were primarily women (16 out of 24). The average age was 47 years, with a range from 32 to 61 years. Three participants worked in the healthcare system at the central level, five at the provincial level, five at the district level, and three in health centers (formally known as small hospitals). Three participants worked for UN agencies, and two worked for NGOs. One was a consultant, and two were active members of civil society associations. Twenty-two interviews were conducted in the Lao language (Table 2).

**Table 2:** Basic characteristics of the participants in the interviews

**Number of participants**		24
**Female**		16
**Age**	mean (min–max)	47 (32–61)
**Organization**	Healthcare institution at central level (Ministry of Health)	3
	Healthcare institution at provincial level (Provincial Health Department)	5
	Healthcare institution at district level (District Health Office)	5
	Health center at the village level	3
	UN agency	3
	NGO	2
	Consultant	1
	Civil society organization	2
**Languages of interview**	Lao (official language)	22
	English	1
	French	1

Eleven documents were analyzed: 1) Fiscal Decentralization in the People’s Democratic Republic of Lao; 2) Decentralization in Lao PDR: A case study of the Effectiveness of Three Build Directive Policy on Local Authority; 3) the role of Marxism in the Lao political system in the contemporary era; 4) the health system review of Lao PDR in 2014; 5) the Prime Minister’s Decree No. 570 on the organizations and operations of the Ministry of Health in 2021; 6) the National Plan of Action on Nutrition (NPAN) 2021–2025; 7) the National Immunization Programme. Updated Comprehensive Multi-Year Plan Lao PDR 2019–2023; 8) Government spending on health in Lao PDR in 2012; 9) National Nutrition Policy in 2008; 10) National Nutrition strategies to 2025 and Plan of Action 2016–2020; and 11) Operational guideline for the implementation of the National Plan of Action on Nutrition (NPAN) 2021 to 2025 [6, 18, 19, 62–69].

### State of decentralization of the Lao healthcare system

**Fig. 2:**
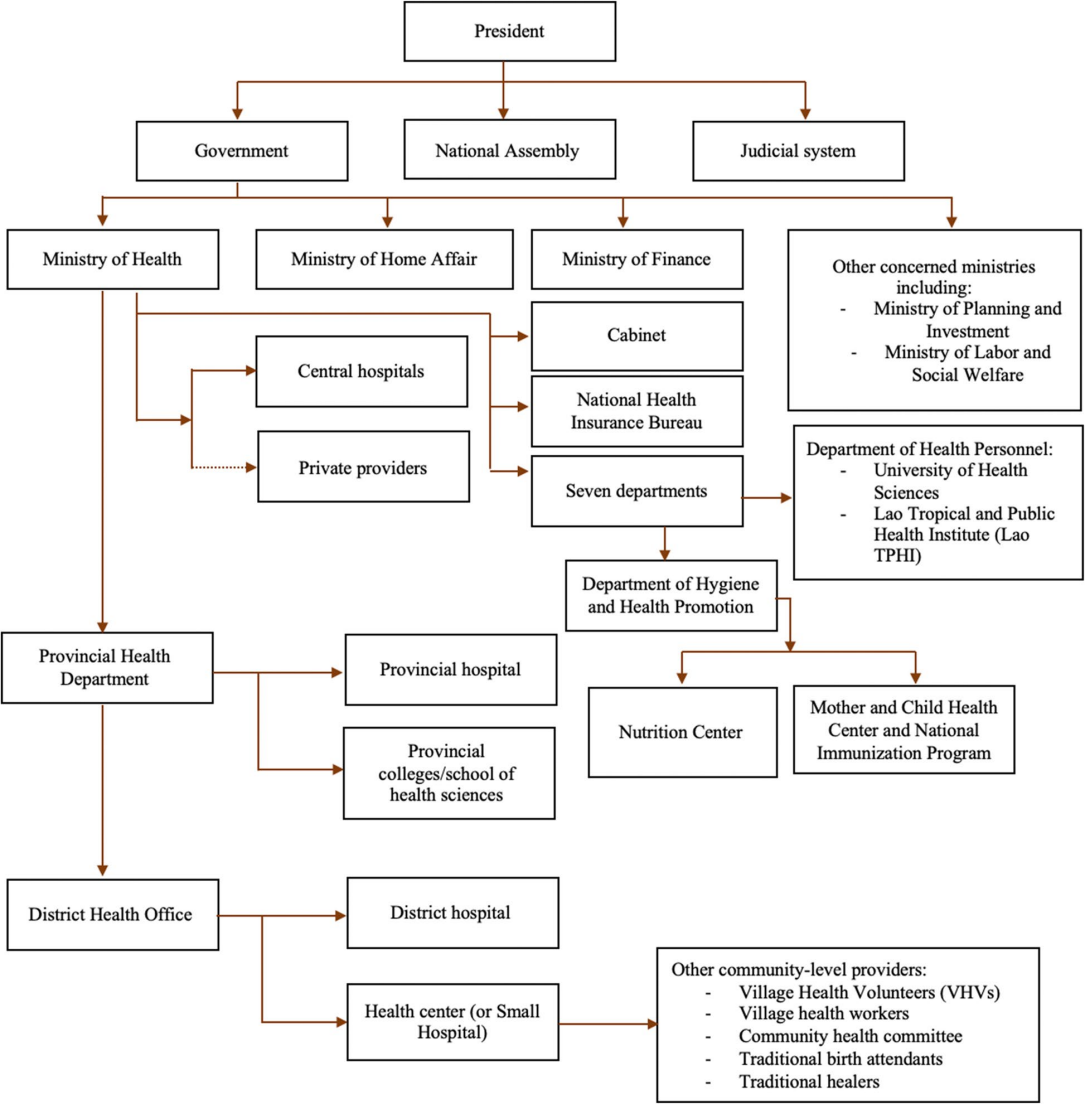
Government-level power sharing in Lao PDR. Sources: Health System Review in Lao 2014; National Plan of Action on Nutrition (NPAN) 2021–2025; National Immunization Programme: Updated Comprehensive Multi-Year Plan Lao PDR 2019–2023; and the Prime Minister’s Decree No. 570 on the organizations and operations of the Ministry of Health in 2021 [6, 63, 64, 68]

Formally, the decentralization of the Lao health system is of the deconcentration type (Fig. 2). The Ministry of Health (MoH) oversees the entire healthcare system, which operates across three levels of governance: central, provincial, and district. Provincial and district health authorities’ main responsibility is to ensure that health facilities comply with centrally defined rules [6, 65]. Private providers must adhere to centrally defined regulations [6].

All decisions concerning human and financial resources in the health care sector must be routed through the central level, represented by the MoH and other ministries, notably the Ministry of Finance (MoF), which is responsible for overall fiscal policy and sectoral allocations of the annual recurrent budget. Other ministries are also involved. The Ministry of Planning and Investment (MPI) oversees capital budget allocation, whereas the Ministry of Home Affairs (MHA) manages the total number of civil servants and sectoral allocations of human resource quotas, including the annual recruitment of health personnel at all administrative levels [6, 65].

Most respondents (19/24) highlighted the fact that in practice, the power dynamics between levels of governance are complex because constitutional power-sharing arrangements are also under political influence. Official documents support this assertion when they emphasize that in the Lao PDR, the Lao People's Revolutionary Party (LPRP) is the body that has the constitutional mandate to exercise leadership across all levels of government [18, 62].

#### Power sharing between government levels and nutrition programs in Lao PDR

The Lao MoH has the ultimate responsibility for uni- and multisectoral nutrition programs. While the Provincial Health Department (PHD) and District Health Office (DHO) are responsible for implementing and ensuring the functionality of services, the central government retains control over which interventions to offer and over the allocation of human, financial, and material resources [66, 69] (Table 3).

**Table 3:** Formal responsibilities ensured by levels of governance in the health sector and specifically for nutrition programs. Sources: Health System Review in Lao 2014; National Plan of Action on Nutrition (NPAN) 2021–2025; National Nutrition Strategy to 2025 and Plan of Action (NNSPA) and Operational guideline for implementation of National Plan of Action on Nutrition (NPAN) 2021–2025 [6, 63, 66, 67]

Administrative levels	Healthcare system	Nutrition field
Central level (MoH)	MoH - Development and implementation of health promotion programs - Responsibility of central and specialist hospitals and health institutes - Provision of human, financial and material resources for all health care facilities in the country - Oversight over curative care and rehabilitation services at all administrative levels - Food and drug safety - Oversight of traditional medicine - Contract with international organizations - Regulation of pharmacy and medicine private sectors	Multisectoral National Nutrition Committee (MSNNC) - Political guidance on nutrition - Coordination between stakeholders at national and subnational levels Nutrition Center (NC) - Technical guidance to all agencies working on nutrition across sectors
Provincial level	Provincial Health Department (PHD) - Advise on health matters to the governor, - Allocation of budget received from the central level to health services - Management of operations under the technical guidance and oversight from the MoH - Responsibility of provincial hospital	Provincial Nutrition Committee (PNC) - Prioritization, and coordination of interventions commended by the MSNNC - Mobilization of stakeholders at the provincial level
District level	District Health office (DHO) - Oversight of district hospitals and health centers, under the head of the district for administrative matters and the PHDs for technical guidance and supervision - Implementation of outreach services to support the health center level centrally defined responsibilities toward communities	District Nutrition Committee (DNC) - Prioritization, and coordination of interventions commended by the PNC - Mobilization of stakeholders at the district level
Village level	- Health centers - Provision of primary healthcare preventive and curative services, under the supervision of the DHO - Oversight over village health volunteers (VHV), who deliver basic care, and facilitate communication between villages and district authorities - Provision of outreach services in the community, such as immunization	Village Nutrition Committee (VNC) - Support to village-level health staff on nutrition interventions, under the supervision of PHD and DHO

At the central level, operational leadership in the field of nutrition is formally led by the Multisectoral National Nutrition Committee (MSNNC), which comprises the Ministry of Health, Ministry of Planning and Investment, Ministry of Agriculture and Forestry, Ministry of Education and Sports, and other ministries and mass organizations. The MSNNC defines priorities, assesses local needs, and oversees the implementation of centrally sanctioned interventions at local levels (Table 3). Although the responsibility for the MSNNC falls under the MoH, the MSNNC is chaired by the Deputy Prime Minister and deputy chaired by the Deputy Minister of Health. Its members consist of deputy ministers of eight ministries and the vice-director or the secretary of the LPRP’s mass organizations. The MSNNC serves as the central committee overseeing the hierarchical structure of other nutrition committees at the provincial, district, and village levels. The MSNNC structure has been conceived with the idea of having leadership able to promote intersectionality of nutrition interventions [6, 63, 64, 66].

Yet, under the authority of the Politburo of the LPRP, the Lao national government holds supreme authority, whereas provincial governments possess financial responsibilities and managerial duties. Provincial administration is directly supervised by the central government under the principle of democratic centralism [18, 19].

### Structure and interpretive schemes underlying the decentralization state of nutrition programs in the Lao PDR

**Fig. 3:**
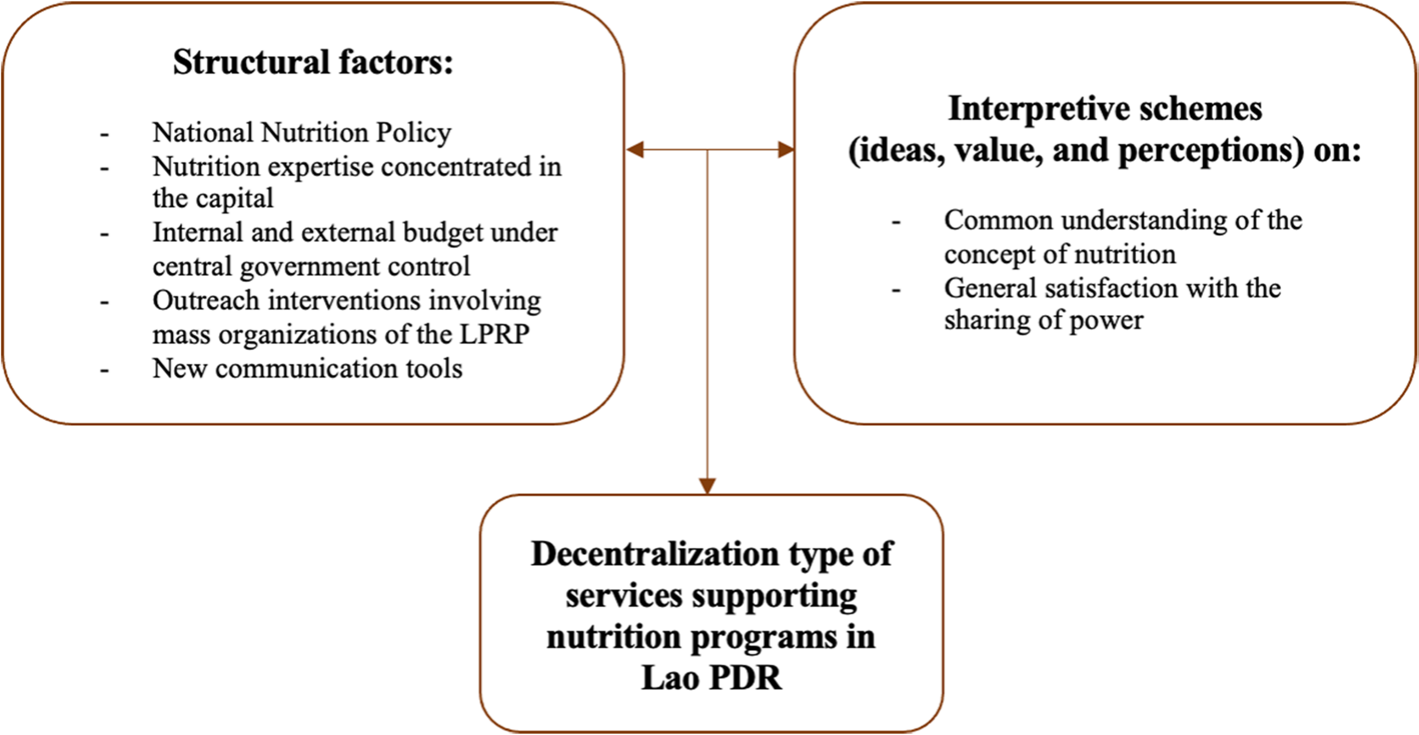
Factors associated with the decentralization type of nutrition programs in Lao PDR

Seven factors related to the structure and interpretative schemes that influence the effectiveness of nutrition programs emerged from the analysis of interviews and documents (Fig. 3).

### Structural factors

Five main structural factors emerge from the data underlying the decentralization state of nutrition programs in Lao PDR: 1) the existence of a public policy; 2) the availability of human resources; 3) the source of funding; 4) the key role of mass organizations; and 5) communication tools.

#### A national nutrition public policy

Interventions on malnutrition in Lao PDR are primarily based on a public policy, the National Nutrition Policy (NNP), which was enacted in 2008. This policy provides the legal framework for mobilizing and coordinating actors that can contribute to improving nutrition indicators, regardless of their sector. The policy is operationalized through formal strategic plans developed by the MoH. These official documents define the responsibilities of agencies addressing malnutrition among the different levels of government in the country [63, 66, 69]. All participants agreed that a public policy on nutrition is essential for ensuring intersectoral collaboration at various levels of governance. The policy is seen as a structural foundation that, while centralizing, guarantees the ability of the concerned players to act.

#### Human resources

The second structuring element that emerges from the data is the fact emphasized by almost all respondents (21/24) and all documents dealing with nutrition [6, 63, 66] that the shortage of expertise outside the capital limits local governments from taking charge of nutrition programs more independently. The respondents also highlighted the fact that a shortage of human resources is compounded by a high turnover rate at all local levels. Turnover weakens the capacity to coordinate the numerous actors involved. Moreover, it drains a considerable part of the local budget, as it leads to continual staff training for nutrition interventions. In brief, all the participants recognized that the state of human resources made it difficult to imagine a program to combat malnutrition run by local authorities.

#### Funding

The health system, from the central to the village levels, is funded from three main sources: a budget allocated by the Ministry of Finance, direct payments at health facilities from users of health services, and funding from external donors, such as NGOs. Funds allocated by the central government are sent to provincial authorities, who then allocate these funds to districts according to planned budgets. These funds are designed for civil servants’ salaries as defined by the central government, as well as for the equipment needs of health establishments. Most of the money paid by service users can be kept by the hospital and used for recurrent costs, including staff wages, allowances, maintenance, utilities, and supplies. The rest (approximately 20%) should be handed over to the provincial or district finance department. Money provided by external donors for projects deployed in provinces and districts must be approved at the central level. One consequence of having multiple sources of financing nutrition activities is the variability in available funds for health services among districts and provinces [6, 18, 65].

The annual budget planning for nutrition interventions involves consultations and reports sent from the district to the provincial levels. The MoH then finalizes and submits it for approval by the National Assembly. In other words, nutrition programs are mainly under central government management. The majority of local-level participants (9/13) feel that this system is not optimal, the main reason being the uncertainty regarding the availability of financial resources over time. These respondents also deplore frequent delays in budget transfers, particularly transfers to remote areas. Above all, nearly all participants at the district and health center levels (6/8) mentioned that nutrition-targeted budgets are often merged with funds intended for non-nutrition public health interventions, as indicated in the following quotation.

“*There’s no other source of money. For the education or agriculture sector, the money would go to each sector separately. For these sectors, they have their own money for nutrition, but we don’t have. So, we need to depend on the integrated work. … Mainly, when we request to the central level, they said they don’t have money for our province anymore; there is only our provincial health office and their help that includes the budget, like they would include the nutrition on the integrated work, and then they manage the staff for it. The structure that divides clearly, it’s not always suitable in real situations*” *said by the participant from the provincial level* (P11)

The irregular flow of funds is partially offset by the availability of external funding, such as from NGOs [63, 66]. However, activities funded by external entities require preapproval from the central government.

The fact that no respondent was able to propose solutions to the problem of funding uncertainty suggests that current funding mechanisms contribute significantly to the solidity of the structure underpinning the decentralization of nutrition programs and hence to the stability of the decentralization type that underlies nutrition programs.

#### LPRP’s mass organizations

In Lao PDR, the LPRP’s mass organizations, particularly Lao Women’s Union (LWU), are unavoidable collaborators in nearly all programs implemented at the population and community levels that aim to reduce the prevalence of malnutrition. Even if these organizations do not necessarily have expertise in health education which is one of their main contributions, there is a consensus among participants that they have proven to be a valuable substitute for the shortage of professionals in the field. This is expressed in the following quotation:

*“…there are the LPRP grassroot members in the meeting (nutrition committee meeting at the district level), who we invited to join us, as well as the standing and district LPRP committee who divides the responsibility for guiding each population group, whoever is responsible for our target group, we would take them to the field, as well as the mass organization, especially the Lao Front for National Development as a main actor to discuss this, second is the Women Union that we can’t forget them, we would take them going to the field with us.” said by the participant from the district level (P17)*

This general feeling is also recorded in several official documents [6, 62, 66].

In short, the constant presence of mass organizations whose primary function, in addition to contributing to health education programs, is to increase LPRP values in the population reinforces the power of the central level in nutrition programs deployed in communities.

#### Communication tools

All the participants underscore the pivotal role of social networks, such as Facebook and WhatsApp, in expediting decision-making processes that previously relied on formal communication channels and informal social gatherings. According to the participants, social media has increased the capacity of nutrition programs to reach their objectives by enabling professionals involved in nutrition programs everywhere to be easily reached and to ask questions without navigating through time consuming and poorly effective bureaucratic procedures. The participants also highlighted that these new technologies inject a sense of dynamism through the competition they foster among teams. This sentiment is captured in the following quotation:

*“In the groups, there are staff (DHO staff) and department level (PHD staff) together... If the department (PHD staff) is not in the group, they will send the responsible staff to join the group. So, we always exchange (information) with each other. Whenever the central level (staff) sends messages in the group, everyone always responds. They couldn’t act like they haven’t seen it (messages) because we have the responsibilities for our work, and they also have their responsibilities. If they don’t answer, it means that they haven’t followed up (on the work)” said by the participant from the central level (P01)*

There is a consensus that new communication tools have reshaped the power dynamics between central and local levels of governance in Lao PDR. They allow overpassing the traditional hierarchical structure of the healthcare system that is supported by communication channels such as those based on the exchange of information by paper (including faxes). This trend empowers lower levels to directly engage with higher-level actors at the central level, fostering a possible emerging capacity for a more decentralized dynamic inside the field of nutrition programs.

### Interpretive schemes

Two main interpretive factors emerged from the data underlying the decentralization state of nutrition programs in Lao PDR: 1) the understanding of the causes and consequences of malnutrition; and 2) the perception of the roles of different levels of government in implementing malnutrition interventions.

#### Understanding the causes and consequences of malnutrition

The participants across different levels of governance in Lao PDR share a common understanding of the causes and challenges of malnutrition. All the participants recognized that malnutrition is a multifaceted issue requiring attention on both the demand and supply sides. They emphasized the fact that in a resource-limited country such as Lao PDR, factors such as a lack of human and financial resources, combined with contextual challenges in vulnerable communities such as poverty, cultural barriers, and food insecurity must all be considered. Additionally, there is a consensus among participants that addressing malnutrition necessitates multisectoral programs, as programs limited to calories and micronutrients may have a limited impact on improving the condition of malnourished children. Numerous other issues, such as water, sanitation, and hygiene (WASH), gender, and the educational levels of parents, have been found to be key determinants of nutrition. They require significant collaboration from different sectors and stakeholders. In short, there is a broad consensus among stakeholders on interpretive schemes.

#### Perception of the capacity of the different levels of governance to assume nutrition responsibilities

The majority of participants across various levels of government (10/16) spontaneously declared that interventions to address malnutrition should be led by the central government, citing the fact that the government has expertise and control over the human, financial and material resources necessary for the programs. It is also recognized that district-level health workers, particularly in remote regions, may lack sufficient training and resources. The remaining six participants did not express an opinion on this matter. Although half of the participants at the local level mentioned that regions are increasingly populated by well-trained healthcare professionals, they believe that this trend cannot have a significant impact on the power sharing arrangements for nutrition programs due to the shortage of human resources, especially in remote regions and because local health volunteers and mass organizations largely compensate for human resource needs.

Therefore, there is no question among participants regarding the state of decentralization of services that underpin nutrition programs in the country.

### Dialectic between structure and interpretive schemes

In brief, there is coherence between the structure and interpretive schemes that underlie the sharing of responsibilities across governance levels in nutrition programs and interventions in Lao PDR. This coherence is reinforced by the respondents' belief that the desired effects of socioeconomic changes, such as an increase in the number of well-trained nutrition professionals and the use of communication tools empowering regional health workers, cannot sufficiently alleviate human resource needs in the provinces. The system of division of responsibilities between governance levels that has lasted for approximately fifty years is therefore likely to be stable. It is likely to remain so for the foreseeable future.

## Discussion

In Lao PDR, powerful forces are pushing the Lao healthcare system toward more centralization than decentralization, despite factors that support greater autonomy for local authorities.

Every organization concerned with nutrition programs seems to be satisfied with this situation and perceives it as a determinant of the effectiveness of nutrition programs. None of the respondents suggested an alternative approach, despite acknowledging that socioeconomic changes are endowing regions with the capacity to manage public health programs that were previously unimaginable. Therefore, there is convergence of interpretive schemes among concerned actors, as well as coherence between structure and interpretive schemes. This convergence signifies a shared vision of nutrition challenges in the country and the capacity to address them, regardless of organizational concerns or hierarchical levels of governance. The coherence between structure and interpretive schemes reveals a general feeling that the current distribution power among governance levels is the most appropriate way to meet the nutritional needs of the population.

From an organizational perspective seen through a neoinstitutional lens [70], the main consequence is that the distribution of responsibilities in nutrition services among different government levels has remained stable since the event of the Lao PDR in 1975.

The findings of this study highlight how a country's sociopolitical context influences the state of decentralization and the functioning of its public health programs. In the case of immunization programs in Lao PDR, three notable factors impact their implementation: the Lao sociopolitical background, the availability of resources, and the use of new technologies such as WhatsApp by health professionals. These elements have reshaped the dynamics of the deconcentrated healthcare system and its program operation [31].

Theoretically, a centralized healthcare system should lead to more standardized public health programs and health services across the country if it ensures consistent application, quality of care, and equitable distribution of resources [71–73]. However, given the diverse contexts among provinces, a one-size-fits-all approach may not be optimal, especially for complex and multisectoral interventions like nutrition programs. These programs often require adaptation to the specific needs and capacities of local contexts and populations in different communities to be truly effective [74, 75].

In a diverse country like Lao PDR, public health interventions require adaptation and more flexible interventions and activities to meet the varied needs of different communities. Empowering local authorities, who are more familiar with local contexts and issues, could improve the management of activities and resources, leading to better interventions that address the specific needs of their populations. In other single-party countries, decentralization has been shown to effectively strengthen public health programs and enhance their effectiveness [76, 77].

The documents and interview findings from this study indicate that the formal structure of the healthcare system in Lao PDR is well defined. The organizational structures of the NNP identify the roles and responsibilities of stakeholders from the central level of the MoH to the implementation level of the village. However, in practice, the implemented programs aimed at reducing the prevalence of malnutrition in children under age 5 in the country are far more challenging than the formal structure suggests [78]. Addressing nutrition outcomes requires a long-term and multifaceted approach, particularly when contributions among actors and power-sharing roles involve local governments working with multiple stakeholders at all levels.

Indeed, Lao PDR faces challenges in reversing the conditions that would allow local governments to put more of their own initiatives into nutrition programs. Unlike in neighboring countries like Vietnam or Thailand, in Lao PDR, the only training on nutrition is a course integrated into a public health master’s program. Additionally, Lao PDR, a country as large as the United Kingdom with a population of 7.7 million inhabitants, is a sparsely populated country. Fifteen of its eighteen provinces have fewer than 500,000 inhabitants. Only one has more than a million [79]. A small population reduces the likelihood of building a critical mass of people from a given province with expertise in nutrition. Although constraints on qualified human resources are common in many low-resource countries [80, 81], these constraints, coupled with the population context in Lao PDR, obviously significantly contribute to the centralization of the Lao healthcare system. In fact, this need for expertise is not limited to nutrition. Optimizing interventions to reduce the prevalence of malnutrition in children under 5 years of age requires tailored approaches that consider the diverse cultural and regional contexts of the population and address multisectoral programs and interventions including food security, water, sanitation, hygiene, maternal education, and social protection. The complexity of these interventions requires multidisciplinary teams [63, 67, 82]. Poorly populated provinces might not be able to gather all this desired expertise. Finally, Lao PDR receives funding for its nutrition program from several external donors and NGOs. This necessitates central-level coordination that might be even more crucial once the country has left, supposedly in 2026, the group of the least developed countries [83]. Leaving this group will have consequences for the support provided by external actors. Expectations are that the central government will assume more responsibilities for its nutrition programs in the near future.

Finally, advancements in communication technologies and the arrival of better-trained human resources offer hope for addressing the shortage of quality human resources that hinder local authorities' leadership in nutrition programs. An increasing number of graduated individuals living in the provinces are proficient in the use of new technologies [84]. This increased utilization of technology is anticipated to further transform traditional barriers that govern interactions among stakeholders. Additionally, interpretive patterns are likely to evolve as professionals in provinces become better informed and more willing to customize nutrition programs to meet the specific needs of local populations. However, owing resource shortages and uneven population distribution in Lao PDR, there are unequal opportunities for local levels to assume more responsibility for nutrition interventions, with little to no chance for decision-making at the local level. This is expected to limit the capacity of sub-national levels from contributing more substantially to nutrition programs than they currently do.

None of the respondents evoked a future where nutrition programs would be more decentralized. Nevertheless, they acknowledged that the current state of decentralization is suboptimal. They recognized that one of the undesirable effects of centralization is the difficulty in adapting services to the specific needs of diverse populations.

## Limitations

This study has several limitations. First, respondents were selected by their organization directors following administrative procedures mandated by research projects in Lao PDR. While this method has likely allowed to capture a diverse range of perspectives on malnutrition, it may have excluded individuals who disagreed with the official discourse. Although the two coders felt that additional interviews would not yield new information, it is possible that additional insights might have been generated by more interviews if soliciting directly potential participants directly could have been done.

Second, as is common in qualitative studies, unconscious subjectivity and bias among researchers may have influenced some interpretations. However, the risk is mitigated by ensuring the validity of four elements of the results, particularly through the triangulation of information from interviews and document reviews, as well as through independent analyses conducted by two researchers.

Finally, the generalizability of the results may be limited. The Lao context is unique, and nutrition programs have specificities that may differ from those of other national health programs functioning in the country. Consequently, the findings of this study may not be directly applicable to other programs or countries, including those with single-party governance structures.

## Conclusion

Decentralization of the Lao healthcare system is of the deconcentrated type, i.e. a system largely under the responsibility of the central government. The transformations taking place in the healthcare system, notably with the use of new information technologies, are changing relationships between individuals across different levels of government and the fact that the provinces are populated by a growing number of professionals trained in nutrition. These factors could lead people to believe that the system would give more responsibility to provincial governments to improve their malnutrition status at local levels. However, the data show that these changes are probably not yet strong enough to change a structure that has endured for approximately fifty years. The *deconcentration* that characterizes decentralization is therefore likely to continue for the foreseeable future.

## Data Availability

The datasets during and/or analyzed during the current study are available from the corresponding author upon reasonable request.

## Abbreviations

CSOs: Civil society organizations
DHOs: District health offices
Lao PDR: Lao People’s Democratic Republic
LPRP: Lao People's Revolutionary Party
LWU: Lao Women's Union
MHA: Ministry of Home Affairs
MSNNC: Multi-sectorial National Nutrition Committee
MoF: Ministry of finance
MoH: Ministry of health
MPI: Ministry of planning and investment
NGOs: Nongovernmental organizations
NNP: National Nutrition Policy
NNSPA: National Nutrition Strategy and Plan of Action
NPAN: National Plan of Action on Nutrition
NIT: Neo-institutional Theory
PHDs: Provincial health departments
UN: United Nations
VHVs: Village health volunteers
WHO: World Health Organization
